# Orthogonal polarisation spectral imaging as a new tool for the assessment of antivascular tumour treatment *in vivo*: a validation study

**DOI:** 10.1038/sj.bjc.6600318

**Published:** 2002-05-03

**Authors:** S Pahernik, A G Harris, M Schmitt-Sody, S Krasnici, A E Goetz, M Dellian, K Messmer

**Affiliations:** Institute for Surgical Research, Klinikum Grosshadern, University of Munich, Marchioninistrasse 15, 81377 Munich, Germany; Department of Anesthesiology, Klinikum Grosshadern, University of Munich, Marchioninistrasse 15, 81377 Munich, Germany; Department of Otorhinolaryngology, Klinikum Grosshadern, University of Munich, Marchioninistrasse 15, 81377 Munich, Germany

**Keywords:** tumour microcirculation, OPS, angiogenesis

## Abstract

Tumour angiogenesis plays a key role in tumour growth, formation of metastasis, detection and treatment of malignant tumours. Recent investigations provided increasing evidence that quantitative analysis of tumour angiogenesis is an indispensable prerequisite for developing novel treatment strategies such as anti-angiogenic and antivascular treatment options. Therefore, it was our aim to establish and validate a new and versatile imaging technique, that is orthogonal polarisation spectral™ imaging, allowing for non-invasive quantitative imaging of tumour angiogenesis *in vivo*. Experiments were performed in amelanotic melanoma A-MEL 3 implanted in a transparent dorsal skinfold chamber of the hamster. Starting at day 0 after tumour cell implantation, animals were treated daily with the anti-angiogenic compound SU5416 (25 mg kg bw^−1^) or vehicle (control) only. Functional vessel density, diameter of microvessels and red blood cell velocity were visualised by both orthogonal polarisation spectral™ imaging and fluorescence microscopy and analysed using a digital image system. The morphological and functional properties of the tumour microvasculature could be clearly identified by orthogonal polarisation spectral™ imaging. Data for functional vessel density correlated excellently with data obtained by fluroescence microscopy (*y*=0.99*x*+0.48, *r*^2^=0.97, *R*_S_=0.98, precision: 8.22 cm^−1^ and bias: −0.32 cm^−1^). Correlation parameters for diameter of microvessels and red blood cell velocity were similar (*r*^2^=0.97, *R*_S_=0.99 and *r*^2^=0.93, *R*_S_=0.94 for diameter of microvessels and red blood cell velocity, respectively). Treatment with SU5416 reduced tumour angiogenesis. At day 3 and 6 after tumour cell implantation, respectively, functional vessel density was 4.8±2.1 and 87.2±10.2 cm^−1^ compared to values of control animals of 66.6±10.1 and 147.4±13.2 cm^−1^, respectively. In addition to the inhibition of tumour angiogenesis, tumour growth and the development of metastasis was strongly reduced in SU5416 treated animals. This new approach enables non-invasive, repeated and quantitative assessment of tumour vascular network and the effects of antiangiogenic treatment on tumour vasculature *in vivo*. Thus, quantification of tumour angiogenesis can be used to more accurately classify and monitor tumour biologic characteristics, and to explore aggressiveness of tumours.

*British Journal of Cancer* (2002) **86**, 1622–1627. DOI: 10.1038/sj/bjc/6600318 www.bjcancer.com

© 2002 Cancer Research UK

## 

The formation of new blood vessels, angiogenesis, is a prerequisite for the growth of solid tumours and forms the basis for new antivascular treatment strategies (Folkman, 1995). Quantitative analysis of tumour vascularity is of paramount importance for the assessment of microvascular function and for the development of novel antivascular treatment strategies. Targeting of the angiogenic pathway to inhibit neovascularisation is a promising new development for the treatment of solid tumours and the prevention of metastasis (O'Reilly *et al*, 1996, 1997; Bergers *et al*, 1999). A prerequisite for monitoring angiogenic activity of tumours *in vivo,* thus investigating the success of such a therapy, is the availability of a quantitative angiogenesis assay (Jain *et al*, 1997).

Currently, however, knowledge about tumour angiogenesis is mainly obtained either by counting the microvessel density of biopsied tumours (Weidner *et al*, 1991) or by immunohistological analysis of angiogenic factors such as VEGF (Gasparini *et al*, 1997). The drawback to these techniques is that they are invasive, one-point measurements and limited to regions of the tumour which are biopsied.

Orthogonal polarisation spectral™ (OPS) imaging is a novel technique which has been introduced recently to visualise microvessels *in vivo* where tissue is illuminated with linearly polarised light and imaged through a polariser oriented orthogonal to the plane of the illuminating light (Groner *et al*, 1999). Using this technique close to wavelengths within the haemoglobin absorption spectrum, microvessels containing red blood cells can be visualised. The OPS imaging system does not require transillumination or fluorescent dyes for contrast enhancement and can be positioned directly on top of the tumour to visualise tumour vessels. The small size of the probe facilitates its use as a non-invasive diagnostic tool not only experimentally but more importantly in clinical settings to evaluate and monitor tumour angiogenesis (Figure 1A

**Figure 5:**
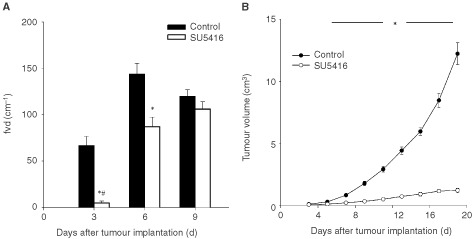
(**A**) Functional microvessel density was assessed by OPS imaging for control and SU5416 treated animals, *P<0.05 SU5416 *vs* control, #*P*<0.05 day 3 *vs* day 6. (**B**) Tumour growth in control and SU5416 treated animals, *P<0.05 SU5416 *vs* control.

**Figure 1:**
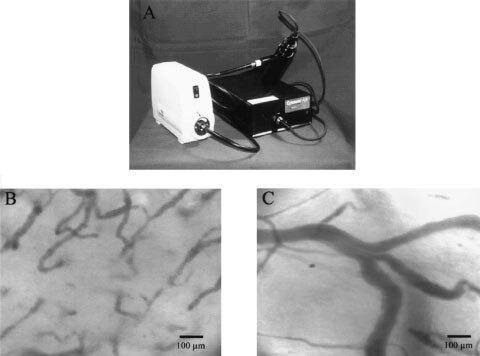
(**A**) Orthogonal polarization spectral (OPS™) imaging probe. (**B**) Representative OPS image of a tongue tumour acquired from a patient. Tumour microvessels can be clearly depicted. (**C**) Representative OPS image of normal tongue microcirculation.

). In fact, images of a human squamous cell carcinoma of the tongue and of normal tongue microcirculation can be obtained using OPS imaging, as shown in Figure 1B,C.

Therefore, it was our aim to evaluate and validate the OPS imaging system with regard to the specific heterogenous tumour vascularity. Animals bearing solid tumours were treated with anti-angiogenic therapy using the synthetic FLK-1 kinase inhibitor SU5416 (Sugen Inc., San Francisco, CA, USA) to block the signal transduction pathway through the VEGF receptor (Fong *et al*, 1999; Vajkoczy *et al*, 1999). Tumour angiogenesis was quantified by OPS imaging and compared with simultaneous intravital fluorescence microscopy investigations for the quantification of tumour angiogenesis and tumour microhaemodynamics. This novel technique allows to quantify noninvasively morphological and functional tumour vascularity. Moreover, OPS imaging allows for assessment of tumour angiogenesis in a clinical setting to assess prognosis of patients and to monitor antivascular and anti-angiogenic treatment regimes.

## MATERIALS AND METHODS

### Animal and tumour model

Following approval of the local ethics committee experiments were performed on male Syrian golden hamsters (50–60 g body weight). The ethical guidelines that were followed meet the standards required by the the UKCCCR ‘Guidelines for the welfare of animals in experimental neoplasia’ (Workman *et al*, 1998). Experiments were performed with male Syrian Golden hamsters (40–60 g body weight) obtained from Charles River (Sulzbach, Germany). They were kept in a normal 12 h light : 12 h dark cycle and fed laboratory chow (ssniff; Spezialdiaeten GmbH, Soest, Germany) *ad libitum* and had free access to drinking water. For the microsurgical procedure, the hamsters were anaesthetised by intraperitoneal injection of ketamine (100 mg kg^−1^ body weight, Ketavet; Parke-Davis, Berlin, Germany) and xylazine (10 mg kg^−1^ body weight, Rompun; Bayer, Leverkusen, Germany). Dorsal skin was shaven and chemically depilated (Pilcamed; Schwarzkopf, Hamburg, Germany). The dorsal skinfold chamber (Asaishi *et al*, 1981; Endrich *et al*, 1982) was implanted by sandwiching the extended double layer of dorsal skin between two symmetrical titanium frames. Twenty-four hours after chamber preparation 2×10^5^ cells of the amelanotic melanoma of the hamster A-Mel-3 were implanted into each chamber (Fortner *et al*, 1961; Endrich *et al*, 1982). After 48 h a fine polyethylene catheter was inserted into the jugular vein for injection of fluorescent dyes.

### Anti-angiogenic treatment

After tumour cell implantation, animals were assigned randomly to two groups. Starting on the day of tumour implantation, animals (*n*=8) were treated daily by intraperitoneal injection of SU5416 twice (Sugen Inc., San Francisco, CA, USA) dissolved in DMSO at a final concentration of 25 mg kg^−1^ body weight. This dosage of SU5416 has been proven to be effective previously in several different animal species (Fong *et al*, 1999). The control animals (*n*=8) received the DMSO only.

### Simultaneous assessment of tumour angiogenesis by intravital microscopy and by OPS imaging

Tumour angiogenesis was quantified at days 3, 6, 9 after tumour implantation. For the experiments, the awake animals were immobilised in a plexiglass tube and the chamber preparation was attached to a microscope stage, which was computer controlled to allow for repeated scanning of identical segments of microvessels. To take advantage of the computer controlled microscope functions the OPS imaging probe was attached to the shaft of the microscope. The plexiglas stage was modified so that there were two different pairs of holes, with which the stage could be attached to the motorised plate. One pair of holes aligned the chamber under the fluorescence microscope and the other set aligned the chamber under the OPS imaging probe. Moving the stage from one pair of holes to the other, the identical region of interest could be observed with the two systems. OPS imaging was using the new Cytoscan E-II instrument (Cytometrics Inc., Philadelphia, PA, USA) (Harris *et al*, 2000) with a final magnification of 462×. The measurement depth of OPS imaging at this magnitude is approximately 300 μm. Validation of OPS imaging was performed with fluorescence microscopy using 100 mg kg^−1^ b.w. fluorescein isothiocyanate-labelled dextran (FITC-dextran, M_r_=500,000 Sigma Chemie, Deisenhofen, Germany) for visualisation of microvessels. For fluorescence microscopy a 20-fold water immersion objective (Zeiss Axiotech 100 HD microscope, Acroplan 20×/0.5 W, Zeiss, Oberkochen, Germany) with a total magnification of 533× was used. A 150 W halogen lamp (Schott 1500, Schott, Mainz, Germany) in combination with a fluorescence filter block (excitation: 450–490 nm, emission >520 nm) was used to visualise FITC-Dextran. Fluorescence images were captured using a charge-coupled device video camera (FK 6990 IQ-S, Piper, Schwerte, Germany). The images from both devices (Cytoscan A/R and fluorescence microscopy) were recorded on S-VHS video tape (video recorder SVO-9500 MDP, Sony, Koeln, Germany) for later off-line analysis.

Six regions of interest (ROI), three from the centre of the tumour and three from the periphery of the tumour, were randomly selected for the assessment of tumour angiogenesis. Analysis of microcirculatory parameters using OPS imaging as well as fluorescence microscopy were performed off-line from the videotapes by digital image analysis system (Cap-Image, Ingeneurbuero Zeintl, Heidelberg, Germany). Functional vessel density (fvd) as parameter of tumour angiogenesis (one per cm), defined as the total length of perfused microvessels per unit area of observation, was quantified (Dellian *et al*, 1996). For microhaemodynamic measurements, the diameter (d) of microvessels (μm) and red blood cell velocity (V_RBC_, mm s^−1^) were measured.

### Evaluation of tumour growth and metastasis formation

For tumour implantation animals were anaesthesised as described above. The dorsal skin of the animals was shaven and depilated. A total of 5×10^6^ cells of the amelanotic melanoma A-MEL-3 suspended in a volume of 10 μl RPMI-1640 medium (Biochrom, Berlin, Germany) were injected subcutaneously in the dorsal skin of hamsters (Kuhnle *et al*, 1992). Tumour diameters, body weight and axillar and inguinal metastasis presence were determined at 2 days intervals beginning at day 3 after tumour implantation. For tumour diameter measurements the longer (l) and shorter (w) perpendicular axes and the height (h) of each tumour were quantified by a caliper (Dellian *et al*, 1994). Individual tumour volume was calculated using the formula *C*×length×weight×height, where *C* has been empirically determined as 0.873, assuming a specific tumour tissue density of 1 g cm^−2^.

### Statistics

Data are expressed as mean±s.e.mean. Nonparametric one-way analysis of variance and multiple comparison on ranks of several independent samples were performed using the Kruskal–Wallis test. Single comparisons of related samples were executed using the Friedman repeated measures on ranks followed by the post-hoc Dunn's test. Independent samples were tested using the Wilcoxon–Mann–Whitney *U*-test followed by the Bonferroni–Holm test. Relation between both techniques was analysed by the Spearman correlation coefficient *R*_s_ and linear regression. Agreement between the two methods was evaluated as bias and precision as described by Bland and Altman (1986). A *P*-value less than 0.05 was regarded as statistically significant.

## RESULTS

### OPS imaging

**Figure 2:**
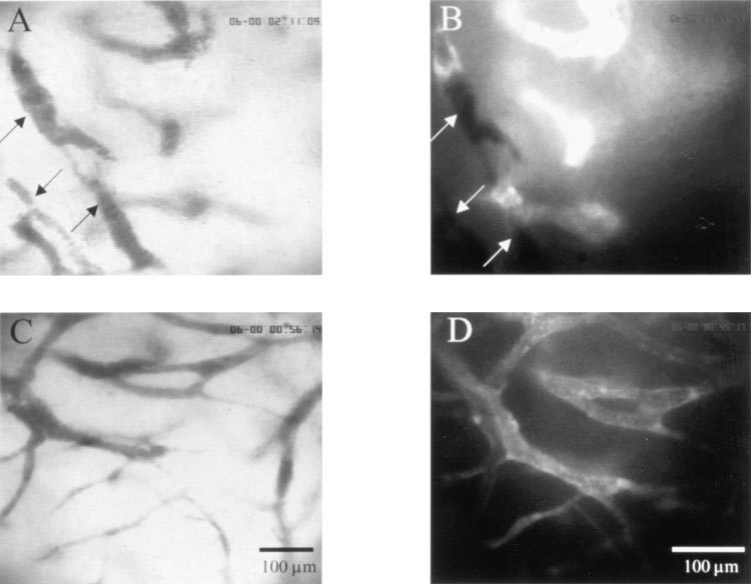
Representative images of tumour microvasculature in animals treated with SU5416 (25 mg kg^−1^ per day, **A**,**B**) and with vehicle (**C**,**D**) at day 6 after tumour cell implantation. Corresponding regions by means of OPS imaging (**A**,**C**) and fluorescence microscopy (**B**,**D**). Arrows indicate non perfused vessels.

The morphological features of the tumour microvasculature were clearly identified by OPS imaging. Figure 2

shows representative corresponding images of the tumour microvessels obtained by OPS imaging (Figure 2A,C) and by intravital fluorescence microscopy using fluorescence labelled dextran as a plasma marker to contrast the microvessels (Figure 2B,D). The vasculature of the tumour is characterised by irregular angio-architecture and heterogeneities of blood perfusion. Tumours of animals treated with SU5416 had reduced angiogenic activity quantified by functional microvessel density (Figure 2A,B), compared to controls (Figure 2C,D).

**Figure 3:**
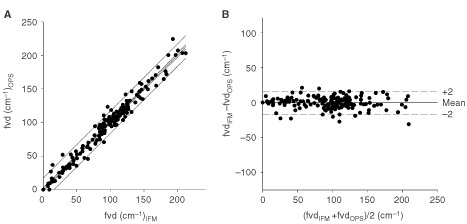
Correlation of functional microvessel density between OPS imaging and fluorescence microscopy and effect of SU5416 on tumour angiogenesis and tumour growth. (**A**) Linear regression analysis between these techniques, lines indicate 95% coinfidence and precision intervals. Correlation parameters for functional microvessel density revealed an excellent correlation for the tumours investigated (*y*=0.99*x*+0.48, *r*^2^=0.97, *R*_S_=0.98, *P*<0.05, *n*=180). (**B**) Agreement between these two identical measurement techniques illustrated as Bland–Altman analysis. Precision and bias calculated by the Bland–Altman analysis between these two measurements techniques was 8.22 cm^−1^ and −0.32 cm^−1^, respectively.

The ability of OPS imaging to analyse tumour angiogenesis was validated by quantifying the functional vessel density, defined as the length of red blood cell perfused capillaries per observation area. Results obtained were compared with data obtained by fluorescence microscopy which is regarded as gold standard for such measurements. Data of functional microvessel density obtained by the two methods revealed an excellent correlation for the tumours investigated (*y*=0.99*x*+0.48, *r*^2^=0.97, *R*_S_=0.98, *P*<0.05, *n*=180, Figure 3A

). Precision and bias calculated by the Bland–Altman analysis between these two measurements techniques was 8.22 and −0.32 cm^−1^, respectively (Figure 3B).

**Figure 4:**
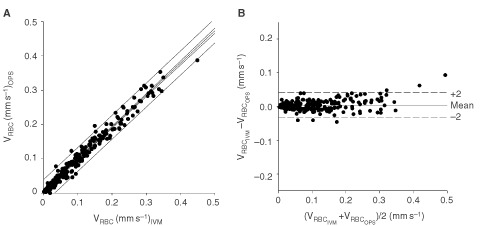
Correlation of red blood cell velocity between OPS imaging and fluorescence microscopy. (**A**) Linear regression analysis between these techniques, lines indicate 95% coinfidence and precision intervals. Correlation parameters for red blood cell velocity revealed an excellent correlation for the tumours investigated (*y*=1.03*x* − 0.0002, *r*^2^=0.97, *R*_S_=0.99, *P*<0.05, *n*=268). (**B**) Agreement between these two identical measurement techniques illustrated as Bland–Altman analysis. Precision and bias calculated by the Bland–Altman analysis between these two measurements techniques was 0.021 mm s^−1^ and a bias of 0.002 mm s^−1^, respectively.

In addition to morphological features, OPS imaging offers the potential to monitor noninvasively and continuously the microhaemodynamics of tumours. Therefore, we compared diameter and red blood velocity of tumour microvessels with data obtained by intravital fluorescence microscopy. The correlations were excellent for measurements of red blood cell velocity (*y*=1.03*x*−0.0002, *r*^2^=0.97, *R*_S_=0.99, *P*<0.05, *n*=268, Figure 4A

) with a precision of 0.021 and a bias of 0.002 mm s^−1^ between both methods (Figure 4B), and for measurements of microvessel diameter (*y*=0.87*x*−1.25, *r*^2^=0.93, *R*_S_=0.94, *P*<0.05, precision: 2.3 μm, bias: 3.5, *n*=268).

### Tumour angiogenesis and microhaemodynamics

Treatment of tumours with SU5416 reduced the formation of new vessels. The functional vessel density of tumours monitored by OPS for control and SU5416 treated animals, respectively, was 66.6±10.1 and 4.8±2.1 cm^−1^ at day 3 after tumour cell implantation (*P*<0.05), 147.4±13.2 and 87.2±10.2 cm^−1^ at day 6 (*P*<0.05), and 119.7±7.2 and 106.1±14.7 cm^−1^ at day 9 (Figure 5A). With respect to red blood cell velocity there were no significant differences between the groups: Red blood cell velocity was 0.09±0.03 and 0.03±0.01 mm s^−1^ at day 3, 0.08±0.02 and 0.11±0.04 mm s^−1^ at day 6, and 0.09±0.02 and 0.07±0.02 mm s^−1^ at day 9 after tumour cell implantation, for control and SU5416 treated animals, respectively. Similarly, no effect of SU5416 treatment on microvascular diameter could be noted. Microvessel diameter was 17.6±2.1 and 12.6±3.8 μm at day 3, 14.5±1.4 and 15.2±1.5 μm at day 6, and 16.6±0.4 and 15.3±2.5 μm at day 9 after tumour cell implantation, for control and SU5416 treated animals, respectively.

### Tumour growth and metastasis development

Exponential tumour growth was observed in all tumours of control animals. In addition to the inhibition of new vessel formation, daily treatment of animals with SU5416 resulted in a significant delay of tumour growth (Figure 5B). At the end of the observation period, 19 days after tumour cell implantation, tumour volume was 12.2±0.9 cm^3^ for control *vs* 1.2±0.2 cm^3^ for anti-angiogenic treated animals. There was no difference in animal body weight over the whole period of observation. Axillary metastases became palpable in all control animals between day 9 and day 11 after tumour cell implantation. In the SU5416 group, only in one animal axillary metastasis formation was palpable at day 19 after tumour cell implantation. Total volume of axillary metastasis at the end of the investigation was 1.09±0.31 *vs* 0.05±0.05 cm^3^ for control and SU5416 treated animals, respectively.

## DISCUSSION

### OPS imaging

OPS imaging is a new technique to visualise microvessels *in vivo*. We have established and validated this new technique for the tumour microvasculature to allow for characterisation of angiogenesis and the effects of anti-angiogenic treatment of tumours. Simultaneous measurements by OPS imaging were compared with intravital fluorescence microscopy investigations of tumour angiogenesis, red blood velocity and vessel diameter. For microvessel density, a well established parameter for tumour angiogenesis (Dellian *et al*, 1996; Jain *et al*, 1997) excellent correlation parameters were found. Furthermore, OPS imaging demonstrated high precision for measurements of red blood cell velocity and microvessel diameter. This issue is of paramount interest because nutritive perfusion not only depends upon the morphological properties of the network of exchange vessels, but also on functional parameters such as red blood velocity and distances between exchange vessels (Intaglietta and Zweifach, 1974). The additional assessment of microhaemodynamic response to antivascular or anti-angiogenic therapies is of major interest when these treatment modalities are combined with different treatment options relying on an intact tumour vasculature such as chemotherapy or radiation therapy (Mauveri *et al*, 1998). However, for measurements of microvessel diameter, parameters assessed for correlation differed. The slope of the linear regression curve was 0.87 with an systematic bias of 3.5 μm indicating an underestimation in the measurements by OPS compared to fluorescence microscopy. This systematic difference is to be expected given the nature of the measurements with fluorescence microscopy. Due to light scattering of the fluorescence light microvessel diameters are overestimated 15% which is in agreement with previous investigations (Gretz and Duling, 1995). Furthermore, streaming of red blood cell velocity in the centre of the vessel potentially contributes to the underestimation of microvessel diameter measurements in OPS imaging. The data are in good aggreement with a previous validation study comparing OPS imaging with intravital microscopy with respect to hepatic microcirculation (Langer *et al*, 2001).

The present study supports SU5416 as a potent synthetic inhibitor of angiogenesis by blocking the VEGF/Flk-1 signal transduction pathway. This compound, which is currently under investigation in Phase III clinical studies for the treatment of human tumours, has been shown to have anti-angiogenic properties and broad antitumour effects leading to a reduction of tumour growth (Fong *et al*, 1999). The inhibition of tumour angiogenesis as quantified by functional vessel density using OPS imaging was accompanied by a sharp reduction in tumour growth and, in the formation of metastasis. However, the inhibition of tumour angiogenesis was only significant on day 3 and 6 after tumour implantation. Other mechanism of tumour angiogenesis than the VEGF mediated signal transduction pathway might lead to the delayed formation of new tumour vessels.

### Implications

OPS imaging technology provides a new method for the assessment of tumour microvessels *in vivo*. The small size of the probe facilitates its use as a non-invasive diagnostic tool in both experimental and clinical settings to evaluate and monitor angiogenesis/angiogenic activity of tumours known to impact the prognosis of the disease. The ability to obtain high contrast images of the vasculature using reflected light will allow for a non-invasive and repetetive quantitative measurement of the distinct morpholgical specifities of the new vasculature such as vascular volume, surface and density as well as quantitative information about the functional characteristics of vasculature with parameters like microvessel density, and vascular dynamics which are both important requirements for angiogenesis assays (Jain *et al*, 1997). The small size and portability of the OPS imaging device compared to normal intravital microscopes makes it possible to use the instrument easily in a clinical setting. As it can be seen in Figure 1B,C, the image quality obtained in a human squamous cell carcinoma of the tongue and in normal tongue microcirculation is comparable to that seen in this study. Quantitative knowledge about angiogenic activity will form the basis for monitoring selective anti-angiogenic and antivascular cancer treatment options in the future.
